# Learning Spatiotemporal Properties of Hippocampal Place Cells

**DOI:** 10.1523/ENEURO.0519-21.2022

**Published:** 2022-07-08

**Authors:** Yanbo Lian, Anthony N. Burkitt

**Affiliations:** Department of Biomedical Engineering, The University of Melbourne, Melbourne, Victoria 3010, Australia

**Keywords:** entorhinal cortex, hippocampus, learning, spatiotemporal properties, sparse coding, theta phase precession

## Abstract

It is well known that hippocampal place cells have spatiotemporal properties, namely, that they generally respond to a single spatial location of a small environment, and they also display the temporal response property of theta phase precession, namely, that the phase of spiking relative to the theta wave shifts from the late phase to early phase as the animal crosses the place field. Grid cells in Layer II of the medial entorhinal cortex (MEC) also have spatiotemporal properties similar to hippocampal place cells, except that grid cells respond to multiple spatial locations that form a hexagonal pattern. Because the EC is the upstream area that projects strongly to the hippocampus, a number of EC-hippocampus learning models have been proposed to explain how the spatial receptive field properties of place cells emerge via synaptic plasticity. However, the question of how the phase precession properties of place cells and grid cells are related has remained unclear. This study shows how theta phase precession in hippocampal place cells can emerge from MEC input as a result of synaptic plasticity, demonstrating that a learning model based on non-negative sparse coding can account for both the spatial and temporal properties of hippocampal place cells. Although both MEC grid cells and other EC spatial cells contribute to the spatial properties of hippocampal place cells, it is the MEC grid cells that predominantly determine the temporal response properties of hippocampal place cells displayed here.

## Visual Abstract

**Figure:**
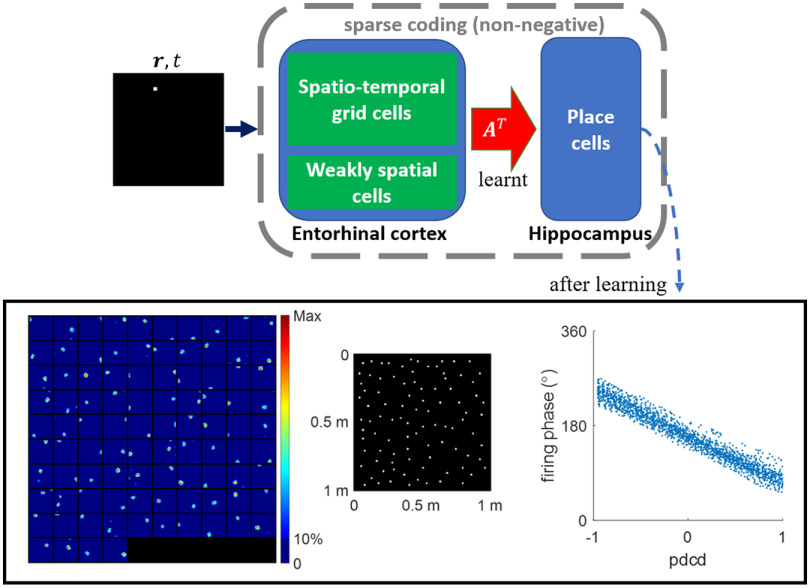

## Significance Statement

In the navigational system of the brain, place cells in the hippocampus and grid cells in the medial entorhinal cortex (MEC) have functionally important spatiotemporal properties. However, little is known about the link between the temporal properties of grid cells and place cells. Recent experimental studies have suggested that temporal properties of hippocampal place cells may be inherited from the MEC. However, a learning model explaining how their relationship can be learnt via synaptic plasticity is still lacking. Here, we build a learning model based on the principle of sparse coding and demonstrate how input from the EC leads to the spatiotemporal properties of hippocampal place cells via synaptic learning.

## Introduction

In early electrophysiological experiments involving freely behaving rats (O’Keefe and Dostrovsky, 1971; O’Keefe, 1976; Hill, 1978; O’Keefe and Conway, 1978), neuroscientists discovered place cells, the principal cells in the hippocampus. Place cells, as suggested by their name, have the spatial property of responding selectively to places in the external environment, namely, they generally respond to a particular location (called the place field) of the spatial environment, although they may respond to multiple locations in a large environment (Park et al., 2011). In addition to the spatial properties of place cells, their temporal response property, namely, theta phase precession, was observed by O’Keefe and Recce (1993): the place cell fires spikes at progressively earlier phases of the local field potential theta rhythm (7–12 Hz) while the animal moves across the place field. Normally, the firing of spike starts at the late phase of the theta rhythm when the animal enters the place field and ends at the early phase when the animal exits the place field (O’Keefe and Recce, 1993; Skaggs et al., 1996).

Three decades after hippocampal place cells were discovered, Hafting et al. (2005) reported another type of spatial cells, the grid cells, in the medial entorhinal cortex (MEC) that is an adjacent area to the hippocampus. Similar to the spatial properties of place cells in the hippocampus, MEC grid cells are also selective to spatial locations of the environment, but each MEC grid cell responds to multiple spatial locations (each location is called a grid field) forming a hexagonal grid that tiles the entire environment (Hafting et al., 2005). Moreover, there is a diversity resulting from multiple hexagonal grids of different MEC grid cells that have different orientations, spacings, and offsets (Stensola et al., 2012). Subsequently, MEC grid cells were also observed to have the temporal response property of theta precession: the firing of MEC grid cells begins at the late phase of the theta rhythm when the animal enters the grid field, the phase of firing shifts in a systematic way during the traversal of the grid field, and ends at the early phase when the animal exits the grid field (Hafting et al., 2008). Although the spatial properties of place cells and MEC grid cells were discovered in an open environment (O’Keefe and Dostrovsky, 1971; Hafting et al., 2005), early studies primarily investigated theta precession on linear tracks (O’Keefe and Recce, 1993; Hafting et al., 2008). The phase precession of place cells and MEC grid cells in the open environment was investigated in later studies by Huxter et al. (2008), Climer et al. (2013), and Jeewajee et al. (2014).

Apart from grid cells in MEC, there are also many nongrid spatial cells in the MEC (Diehl et al., 2017; Hardcastle et al., 2017). In the lateral EC (LEC), many cells also contain spatial information (Hargreaves et al., 2005; Yoganarasimha et al., 2011). In this paper, we refer to these cells in the EC that contain spatial information but display no spatial structure like grid cells as EC weakly spatial cells.

Hippocampal place cells and MEC grid cells are fundamental units of the navigational system of the brain and there is a close relationship between these two types of cells. Experimental studies indicate that input from the EC is the principal input to the hippocampus (Steward and Scoville, 1976; Tamamaki and Nojyo, 1993; Leutgeb et al., 2007; Zhang et al., 2013). Consequently, MEC grid cells along with other cells in the EC are thought to provide spatial input for hippocampal place cells, so that they can have a specific place tuning. This has led to numerous feedforward EC-hippocampus models in which single place fields are generated from EC input, such as MEC grid cells or EC weakly spatial cells (Rolls et al., 2006; Solstad et al., 2006; Franzius et al., 2007a,b; de Almeida et al., 2009; Hasselmo, 2009; Savelli and Knierim, 2010; Neher et al., 2017; Lian and Burkitt, 2021). Furthermore, Fiete et al. (2008) showed that this MEC grid cell structure represents an encoding scheme for position that is analogous to the residue number system (Soderstrand et al., 1986), which is a highly efficient and accurate method for place representation (Sreenivasan and Fiete, 2011). On the other hand, place cells in CA1 of the hippocampus project back directly or indirectly to the EC (Naber et al., 2001; Kloosterman et al., 2003; Slomianka et al., 2011) and the inactivation of place cells leads to the degradation of receptive field structure of MEC grid cells (Bonnevie et al., 2013), supporting a loop-like EC-hippocampus network structure. This loop-like structure has been adopted in some modeling studies to explain other spatial properties of place and MEC grid cells such as global and rate remapping of place cells, and multisensory integration in MEC grid cells (Rennó-Costa and Tort, 2017; Li et al., 2020). Compared with the feedforward EC-hippocampus models, the loop-like models can also incorporate the effects on the spatial firing of MEC grid cells because of the feedback introduced in such models.

Separate from the link between the spatial properties of place cells and MEC grid cells, some experimental studies also infer a link between the temporal properties of place cells and MEC grid cells. Hafting et al. (2008) discovered that MEC grid cells have hippocampus-independent phase precession and suggested that phase precession of hippocampal place cells could be inherited from the EC. Yamaguchi et al. (2007) proposed a entorhinal-hippocampal network that hypothesizes that theta phase precession originates at EC superficial layers and is transmitted to the hippocampus along the hippocampal trisynaptic circuit. In a review paper, Bush et al. (2014) proposed that theta phase precession of place cells is likely inherited from MEC grid cell input. In a later study by Schlesiger et al. (2015), the MEC was found to be necessary for the temporal properties of hippocampal neuronal activity, even when place cells maintain stable spatial firing. Jaramillo et al. (2014) built a computational model that demonstrates that theta phase precession in the EC can propagate to the downstream such as CA1 in the hippocampus. However, unlike many EC-hippocampus models that explain how spatial properties of hippocampal place cells can be learnt using EC input, there are no existing EC-hippocampus learning models that explicitly show how the temporal properties of hippocampal place cells can be inherited from MEC grid cells through some form of Hebbian learning. We propose here a learning model in which the spatiotemporal properties of hippocampal place cells emerge through synaptic plasticity during navigation of a virtual rat in a 2D environment.

The learning model presented here is built on our previous work that shows that the model based on non-negative sparse coding can learn an efficient hippocampal place map using EC input such as MEC grid cells and EC weakly spatial cells (Lian and Burkitt, 2021). In this paper, building on the work of Jeewajee et al. (2014) and McClain et al. (2019), we construct a mathematical model of MEC spatiotemporal grid cells of a 2D environment. These MEC spatiotemporal grid cells, along with EC weakly spatial cells, form the input to modelled hippocampal cells, and the connection between EC input cells (MEC grid cells and EC weakly spatial cells) and modelled hippocampal cells are learnt during navigation of a virtual rat. After learning, the modelled hippocampal cells display spatial and temporal properties that are similar to experimental data of place cells. Furthermore, after the learning process, these learnt hippocampal place cells still maintain their spatial selectivity even when MEC spatiotemporal grid cells are inactivated, suggesting that the remaining EC weakly spatial cells can maintain the place field responsiveness of place cells. Combining our previous work showing that either MEC grid cells or EC weakly spatial cells can give rise to the spatial tuning of hippocampal place cells (Lian and Burkitt, 2021), we complete the picture of how EC input contributes to the properties of hippocampal place cells from the perspective of synaptic plasticity, namely, that hippocampal place cells learn the spatial properties from both MEC grid cells and EC weakly spatial cells, and learn their temporal properties from MEC grid cells. The synaptic plasticity underlying the learning model here is based on the principle of sparse coding.

## Materials and Methods

### The environment and trajectory

The spatial environment used in this study is a 1m × 1m square box where a virtual rat runs freely. Similar to the study by D’Albis and Kempter (2017), the running trajectory $r_{t}$ is generated from the stochastic process described as

(1) $$\frac{\text{d}r_{t}}{\text{d}t}=v_{t}\left[\cos(\theta_{t}),\sin(\theta_{t})\right]\text{ with }\theta_{t}=\sigma_{\theta }\omega_{t},$$

where *v_t_* is the speed sampled from an Ornstein–Uhlenbeck process with long-term mean ${\bar{v}}_{t}=v$, *θ_t_* is the direction of movement, *ω_t_* is a standard Wiener process, and *σ_θ_* is the parameter that controls the tortuosity of the running trajectory.

When the virtual rat is running toward the wall and very close to the wall (within 2 cm), the running direction of the rat (*θ_t_*) is set to the direction parallel to the wall. If the rat location generated by Equation 1 falls outside of the environment, the stochastic process keeps running until a valid location is generated.

The running trajectory of the virtual rat is generated at 100 Hz, i.e., the position is updated every 10 ms according to Equation 1. The long-term mean speed, *v*, is set to 30 cm/s. A summary of parameters and values used in the simulation can be found in Table 1.

**Table 1:** Model symbols and parameters

Description (value)	Symbol
Tortuosity of the running trajectory (1 radian)	*σ_θ_*
Spatial location/running speed/running direction and at time *t*	$r_{t}$/*v_t_*/*θ_t_*
Long-term mean of the speed (30 cm/s)	*v*
Time step of the running trajectory (10 ms)	
Duration of the training/testing running trajectory (3600/1200 s)	
Spatial location in the environment	***r***
Spatiotemporal response at spatial location ***r*** at time *t*	f(r,t)
Spatial receptive field	$f_{s}(r)$
Amplitude/center/radius of the spatial receptive field	*α_c_*/ $r_{c}$/*R*
Phase modulation at spatial location ***r*** at time *t*	$f_{\phi }(r,t)$
Level of phase modulation (uniformly sample from [0.8 1.2])	*k_ϕ_*
Frequency of the theta rhythm (10 Hz)	*F*
Firing phase at location ***r***	ϕ(r)
Entry phase of phase precession (uniformly sampled from [300° 340°])	*ϕ*_0_
Phase change of phase precession (uniformly sampled from [300° 340°])	Δ*ϕ*
Projected center/radius of the spatial receptive field onto running direction	r′/*R*′
Fitted spatial receptive fields of modelled hippocampal cells	${\hat{f}}_{s}(r)$
Amplitude/center/radius of the fitted spatial receptive field	${\hat{\alpha }}_{c}$/ ${\hat{r}}_{c}$/ $\hat{R}$
Fitted level of phase modulation	${\hat{k}}_{\phi }$
Fitted frequency of theta rhythm	$\hat{F}$
Fitted phase of the temporal response	$\hat{\phi }$
Membrane time constant of modelled hippocampal cells (10 ms)	*τ*
Membrane potentials of modelled hippocampal cells	***u***
Firing rates of modelled hippocampal cells	***s***
Firing rates of EC input cells	***I***
Excitatory connection: EC input cells to modelled hippocampal cells	**A**
Firing threshold of modelled hippocampal cells (0.3)	*β*
Learning rate (0.01)	*η*
Time step of computing modelled hippocampal cells by Equation 7 (0.2 ms)	
Number of modelled hippocampal cells in scenarios 1, 2, and 3 (100)	
Number of MEC spatiotemporal grid cells in scenarios 1, 2, and 3 (900)	
Number of EC weakly spatial cells in scenarios 2 and 3 (400)	

### Model of MEC spatiotemporal grid cells

MEC grid cells are found to have spatial and temporal properties, namely, they are selective to a hexagonal grid of spatial locations (Hafting et al., 2005) and have diverse grid parameters (Stensola et al., 2012), and their firing displays theta phase precession (Hafting et al., 2008). The spatial receptive fields of MEC grid cells can be characterized by the sum of three sinusoidal gratings (Solstad et al., 2006) or the sum of circular fields at different locations in the environment. In order to investigate the question of whether the spatial and temporal properties of hippocampal place cells can be inherited from MEC grid cells via learning, we first need a mathematical model that can characterize both spatial and temporal properties of MEC grid cells.

Burgess (2008) designed a model of grid cells based on the oscillatory interference model (O’Keefe and Burgess, 2005; Burgess et al., 2007). This model of grid cells consists of multiple membrane potential oscillators whose frequencies linearly depend on the speed and can capture the spatial and temporal properties of the grid cells. However, a recent experimental study challenged the classical view that the oscillation frequency linearly depends on the running speed (Kropff et al., 2021). Instead, the frequency of the theta rhythm is controlled by the acceleration (Kropff et al., 2021). In this study we build a mathematical model of spatiotemporal grid cells from a different perspective, inspired by the work of Chadwick et al. (2015) and McClain et al. (2019), in which the spatiotemporal responses of grid cells are modelled as the product of spatial and temporal responses as described below.

Chadwick et al. (2015) and McClain et al. (2019) build a mathematical model for 1D place cells that have spatial and temporal properties, and Jeewajee et al. (2014) showed that theta phase precession of grid cells and place cells strongly depends on the projected distance on the current running direction (pdcd) in a 2D environment. Analogous to their work, we construct a mathematical model of MEC spatiotemporal grid cells based on 2D spatiotemporal place cells. The fundamental idea is that the receptive field of each MEC spatiotemporal grid cell is seen as the combination of multiple grid fields located in the hexagonal grid where each grid field has spatiotemporal properties similar to a 2D spatiotemporal place cell.

Within the region of any receptive field, the firing rate at location ***r*** and time *t* is modelled as the product of a spatial receptive field and a phase modulation (McClain et al., 2019):

(2) $$f(r,t)=f_{s}(r)\cdot f_{\phi }(r,t).$$

**Figure 1.**
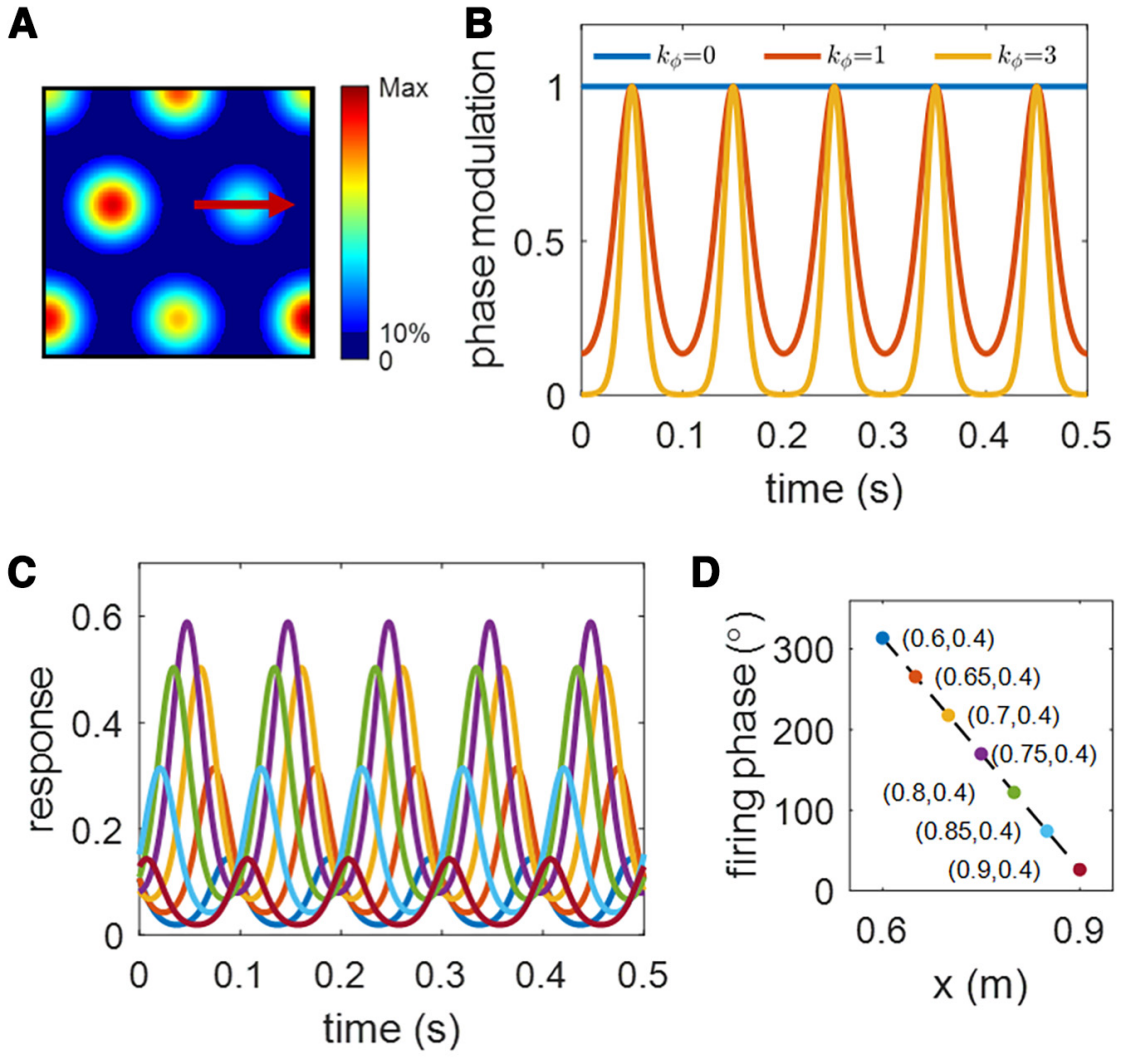
Illustration of the mathematical model of a MEC spatiotemporal grid cell. For this cell, parameters that characterize the cell are: *k_ϕ_* = 1, $\phi_{0}=320^{{}^{\circ}},\;\Delta \phi =300^{{}^{\circ}}$, *R *=* *0.16 m and *F *=* *10 Hz. ***A***, The spatial receptive field (rate map), $f_{s}(r)$, in Equation 3, of a MEC grid cell. ***B***, Phase modulation, $f_{\phi }(r,t)$, in Equation 4, for three different values of *k_ϕ_*. ϕ(r) is set to 180°. The response is more phase locked for larger values of *k_ϕ_*. ***C***, Curves in different colors represent responses, f(r,t), of one prebuilt spatiotemporal grid cell over 0.5 s at different locations (coordinates found in plot ***D*** with the same color coding) when the virtual rat moves from left to right of the place field along the trajectory in plot ***A***. ***D***, Firing phase, ϕ(r), versus position. Only *x* varies because the animal moves straight from left to right.

The spatial receptive field, $f_{s}(r)$, is described as a 2D function:

(3) $$f_{s}(r)=\alpha_{c}e^{-\ln\;5\frac{||r-r_{c}|{|}^{2}}{R^{2}}},$$

where $r_{c}$ is the center of the receptive field, *α_c_* is the amplitude at the center, and *R* determines the radius of the spatial receptive field. Similar to our previous work (Lian and Burkitt, 2021), the spatial receptive field of each MEC spatiotemporal grid cell is determined by randomly sampling the grid spacing, orientation and offset from some distributions. Stensola et al. (2012) showed that MEC grid cells are discretized into modules based on their grid spacings. Following the model of Neher et al. (2017), we take four discrete grid modules whose mean grid spacings are 38.8, 48.4, 65, and 98.4 cm, and mean grid orientations are 15°, 30°, 45°, and 0°, respectively. Similar to the distribution of grid spacings in discrete grid modules (Stensola et al., 2012), these four discrete grid modules account for 43.5%, 43.5%, 6.5%, and 6.5% of grid cells in the model, respectively. For any MEC grid cell in its module, the grid spacing is sampled from a normal distribution with mean spacing and standard deviation of 8 cm, the grid orientation is sampled from a normal distribution with mean orientation and standard deviation of 3°, and the grid offset is sampled from a uniform distribution between 0 and the grid spacing. Because Ismakov et al. (2017) observed the variability in individual grid fields of a MEC grid cell, the amplitude (*α_c_* in Eq. 3) of each grid field for a MEC grid cell is sampled from a normal distribution with mean 1 and standard deviation 0.1 (Neher et al., 2017; Lian and Burkitt, 2021). The radius, *R* in Equation 3, of the receptive field is determined by *R *=* *0.32*λ*, where *λ* is the grid spacing of MEC grid cell. The spatial receptive field (rate map) of one example MEC grid cell is displayed in Figure 1*A*.

The phase modulation, $f_{\phi }(r,t)$, describes the temporal response (i.e., the probability of the timing of the individual action potentials) and is given by

(4) $$f_{\phi }(r,t)=e^{k_{\phi }\left(\cos(2\pi Ft-\phi (r))-1\right)},$$

where *k_ϕ_* is the parameter that controls the level of phase modulation and hence the extent of the resulting phase precession, *F* is the frequency of theta rhythm and set to 10 Hz throughout the paper. The choice of *F *=* *10 Hz help with visualizing the results as every 0.1 s is a full period (see examples in Fig. 1*B*,*C*) and other choices of *F* will not change the results, apart from the frequency of the theta rhythm. ϕ(r) is a function that returns the firing phase at location ***r***. Figure 1*B* shows the dependence of phase modulation on *k_ϕ_*. ϕ(r) is set to 180° when plotting the curve. The larger *k_ϕ_* is, the stronger the phase modulation is. When *k_ϕ_* is 0, there is no phase modulation and the response of MEC grid cells only depends on the spatial location of the animal.

In a 1D linear track, Chadwick et al. (2015) and McClain et al. (2019) model *ϕ*(*r*) as a linear function of the location, *r*:

(5) $$\phi (r)=\phi_{0}-\Delta \phi \frac{r-r_{c}+R}{2R},$$

where *ϕ*_0_ is the entry phase at location *r_c_* – *R*, Δ*ϕ* is the phase change across the receptive field, and thus the exit phase at location *r_c_* + *R* is *ϕ*_0_ – Δ*ϕ*. Note that in 1D *r* and *r_c_* become scalars instead of vectors. An example of the linear relationship between *ϕ*(*r*) and *r* described in Equation 5 is shown in Figure 1*D*.

However, determining ϕ(r) is more complicated in a 2D environment because the animal can enter and exit the receptive field from any location with any running direction, and the running trajectory does not always pass through the center of the receptive field. Jeewajee et al. (2014) show that the best correlate of phase precession in a 2D open environment is the projected distance onto the animal’s current running direction (pdcd). Therefore, we model ϕ(r) in a 2D environment by a projected version of *ϕ*(*r*) in Equation 5, described by:

(6) $$\phi (r)=\phi_{0}-\Delta \phi \frac{r-r_{c}\prime +R\prime }{2R\prime },$$

where $r_{c}\prime$ is the projected center and *R*′ is the projected radius onto the running direction. In any location within the receptive field, the firing phase ϕ(r) depends on both the spatial location and running direction. Firing phases at the same location of different trajectories could have different values because of different running directions.

Above all, the spatiotemporal responses of a MEC grid cells in a 2D environment are simply modelled as multiple grid fields at all the vertices of the hexagonal grid in which each grid field is characterized by the spatiotemporal model described above (Eqs. 2–4, 6).

From Equation 4, we can see that the spatiotemporal response is periodical with frequency *F* when the virtual rat stays at a fixed location within the receptive field. Therefore, supposing the virtual rat remains stationary at ***r*** for 1 s, the spatiotemporal response over this 1 s will oscillate at frequency *F* with phase determined by ϕ(r) and the magnitude of the oscillating response is determined by $f_{s}(r)$. In other words, from the spatiotemporal response over 1 s at a fixed location, we can infer the firing phase and firing frequency of the temporal coding at the location. Although theta waves are generally absent in stationary animals (Bland, 1986), the spatiotemporal firing response over 1 s at a fixed location is used in this paper to visualize, estimate and analyze the temporal properties at different locations within the receptive field. However, this does not imply that the virtual rat stays at any location for 1 s when freely exploring the environment. When the model is in the learning stage, the virtual rat is continually moving in a spatial environment along a trajectory generated by Equation 1. After the learning stage, the spatiotemporal response over 1 s at different locations is used to recover the learnt temporal properties of hippocampal place cells (details found below, Fitting the spatiotemporal response of learnt hippocampal place cells, and Measuring the temporal properties of learnt hippocampal place cells).

The response of one example MEC spatiotemporal grid cell is illustrated in Figure 1. The spatial receptive field (rate map) of this grid cell is displayed in Figure 1*A*. For this cell, $k_{\phi }=1,\;\phi_{0}=320^{{}^{\circ}},\;\Delta \phi =300^{{}^{\circ}}$, and *R *=* *0.16 m. Figure 1*A*, red arrow, indicates the straight trajectory the virtual rat runs through a grid field from left to right. Figure 1*C* shows the response over 0.5 s when the virtual rat is at different locations of the running trajectory (Fig. 1*A*, red arrow) in which the coordinates of locations are given in Figure 1*D* with the same color coding. At each location, the response over 0.5 s is intentionally plotted to visualize the spatial and temporal properties. As the virtual rat runs from the left to the right of the grid field, the spatial response ( $f_{s}(r)$ in Eq. 3), describing the amplitude of the periodic curve, increases, peaks at the center, and then decreases. However, the phases of these periodic curves (Fig. 1*C*) start with a value close to 360° and then keep decreasing along the trajectory, mimicking theta phase precession. Theta phase precession can also be observed from Figure 1*D*, which shows that the curve of firing phase versus position (10) has a correlation coefficient −1. Note that theta phase precession is incorporated into the model through the temporal way in which the firing phase varies linearly with the pdcd within the receptive field (Eqs. 4, 6). In this way, Figure 1 illustrates that our mathematical model of MEC spatiotemporal grid cells shows both spatial and temporal dependence, and the theta phase precession introduced here is perfect with a correlation coefficient −1. Naturally, the theta phase precession is unlikely to be so perfect in experimental studies. However, our aim here is to investigate from a modeling perspective how well the modelled hippocampal place cells preserve the theta phase precession of MEC spatiotemporal grid cells after learning.

### EC weakly spatial cells

Although MEC grid cells have a clear spatial structure, they account for only ∼20% of the MEC cell population (Diehl et al., 2017). Furthermore, Diehl et al. (2017) found that about two-thirds of MEC cells (i.e., the nongrid spatial cells) have less specialized but consistent spatial firing patterns. Hardcastle et al. (2017) discovered that many nongrid cells in the MEC have firing patterns that contain spatial information. In addition, cells in LEC, that also contain spatial information (Hargreaves et al., 2005; Yoganarasimha et al., 2011), can likewise contribute to the formation of hippocampal place cells. In our previous work, we have shown that a model based on sparse coding can effectively retrieve place information from EC weakly spatial cells that have no structured spatial selectivity to form an efficient hippocampal place map (Lian and Burkitt, 2021). In this paper, we incorporated EC weakly spatial cells and treated them as potential upstream input to the hippocampus to investigate how EC weakly spatial cells and MEC spatiotemporal grid cells altogether contribute to the spatial and temporal properties of modelled hippocampal place cells. In this model, the firing field of EC weakly spatial cells is generated by first sampling from a uniform distribution between 0 and 1 for each location, then smoothing the map with a Gaussian kernel with a standard deviation of 6 cm, and normalizing the map to give values between 0 and 0.1.

### Non-negative sparse coding

Similar to our previous work (Lian and Burkitt, 2021), we build here the learning model of spatiotemporal place cells based on non-negative sparse coding (Hoyer, 2003). Sparse coding, originally proposed by Olshausen and Field (1996, 1997), finds an efficient representation of the input using a linear combination of some basis vectors. Non-negative sparse coding constrains the responses and basis vectors to be non-negative. We implement the model via a locally competitive algorithm (Rozell et al., 2008) that efficiently solves sparse coding as follows:

(7) $$\begin{array}{l} \tau \dot{u}=-u+A^{T}I-Ws\\ s=\max(u-\beta ,0) \end{array}$$

and

(8) $$\Delta A=\eta (I-As)s^{T}\text{ with }A\geq 0,$$

where **I** is the input, **s** represents the response (firing rate) of the model units, **u** can be interpreted as the membrane potential, **A** is the matrix containing basis vectors and can be interpreted as the connection weights between the input and model units, $W=A^{T}A-1$ and can be interpreted as the recurrent connection between model units, 1 is the identity matrix, *τ* is the time constant, *β* is the positive sparsity constant that controls the threshold of firing, and *η* is the learning rate. Each column of **A** is normalized to have length 1. The non-negative constraints are incorporated into the system, as seen from the non-negativity of both **s** and **A** in Equations 7 and 8. The parameters of implementing Equations 7 and 8 are given below (see below, Training). Additional details about the implementation of non-negative sparse coding can be found in Lian and Burkitt (2021).

### Structure of the model

**Figure 2.**
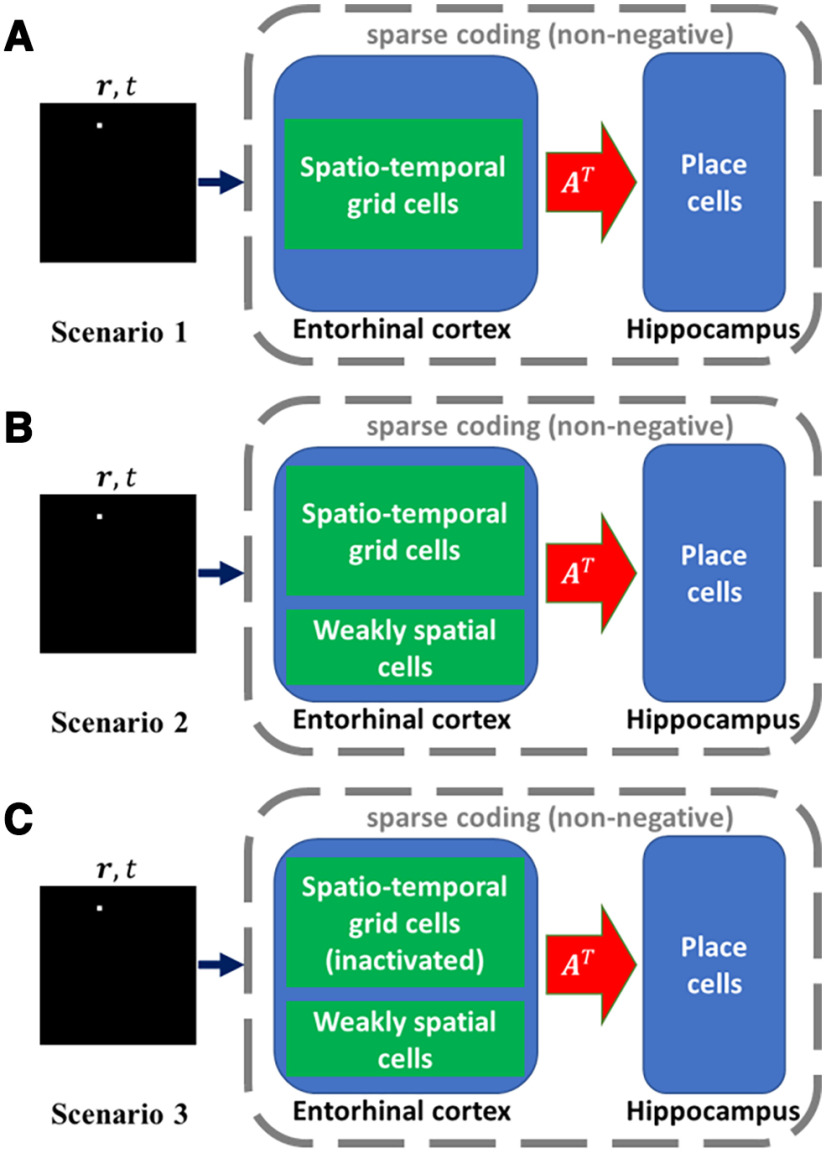
Model structure in three scenarios. MEC spatiotemporal grid cells or EC weakly spatial cells serve as the upstream input to the hippocampus. Given a spatial location ***r*** from a virtual rat running trajectory and time *t*, EC provides input for the hippocampus. The response of modelled hippocampal cells and the connection weights A are updated as described by Equations 7 and 8. ***A***, Scenario 1. Only MEC spatiotemporal gird cells serve as the EC input. ***B***, Scenario 2, Both MEC spatiotemporal grid cells and EC weakly spatial cells serve as the EC input. ***C***, Scenario 3. MEC spatiotemporal grid cells were inactivated after the learning process of scenario 2.

In this paper, the EC provides upstream input for the hippocampus and the EC-hippocampus pathway is modelled in three scenarios. Modelled hippocampal cells undergoes a learning process described by Equations 7 and 8. A modelled hippocampal cell is referred to as “learnt hippocampal place cell” if it meets the criteria of a place cell after learning (see below, Selecting place cells). The diagram of the model structure is displayed in Figure 2. A summary of all symbols defined in this paper is shown in Table 1.

#### Scenario 1

Modelled hippocampal cells only receive input from MEC spatiotemporal grid cells, as described above, which have both spatial and temporal response properties (see above, Model of MEC spatiotemporal grid cells). The EC-hippocampus connection undergoes a learning process while the virtual rat is exploring the environment. This scenario is designed to investigate the contribution of MEC spatiotemporal grid cells to the spatial and temporal properties of learnt hippocampal place cells.

#### Scenario 2

Modelled hippocampal cells receive input from MEC spatiotemporal grid cells together with EC weakly spatial cells. Similar to scenario 1, the EC-hippocampus connection is learnt. This scenario aims to validate and extend the results of scenario 1 to the situation in which other spatial cells in the EC also contribute to the firing of learnt hippocampal place cells.

#### Scenario 3

After the learning process of scenario 2, MEC spatiotemporal grid cells are inactivated and there is no learning in this scenario. This scenario is used to replicate the experimental setup of the study by Schlesiger et al. (2015), in which rats received NMDA lesions of the MEC. Schlesiger et al. (2015) found that theta phase precession of hippocampal place cells was greatly disrupted by MEC lesions but hippocampal spatial firing still remained. This scenario is also similar to another experimental study that showed the retention of hippocampal place fields during medial septum inactivation (Brandon et al., 2014), which is presumed to shut off grid cell input because of the loss of excitatory drive (Brandon et al., 2011; Koenig et al., 2011). In this scenario, the spatial firing of learnt hippocampal place cells will be investigated after the inactivation of MEC spatiotemporal grid cells to compare the simulation results of our model with the results of these experimental studies.

### Training

The values of training parameters and definition of symbols can be found in Table 1. As the virtual rat moves in the environment, each MEC spatiotemporal grid cell generates a firing rate that is determined by the spatial location ***r*** and time *t* according to Equations 2–4 and 6, while each of the EC weakly spatial cells generates a firing rate determined only by the spatial location. The response of modelled hippocampal cells is computed iteratively using the model dynamics (Eq. 7). The connection, **A**, between EC cells and modelled hippocampal cells is updated according to the learning rule described in Equation 8. There are 100 modelled hippocampal cells in the model and the three different scenarios above are implemented.

#### Scenario 1

Only MEC spatiotemporal grid cells provide input to the modelled hippocampal cells, there are 900 MEC spatiotemporal grid cells, i.e., **A** is a 900 × 100 matrix. A running trajectory of 3600 s is used to train the model and the connection weight matrix, **A**, is learnt during this training process.

#### Scenario 2

Both MEC spatiotemporal grid cells and EC weakly spatial cells provide input to the hippocampus. In addition to the 900 MEC spatiotemporal grid cells, there are also 400 EC weakly spatial cells, i.e., **A** is a 1300 × 100 matrix where the top 400 rows of **A** represent the connection weights from EC weakly spatial cells to modelled hippocampal cells and the bottom 900 rows represent the connection weights from MEC grid cells to modelled hippocampal cells. The same running trajectory of 3600 s, as used in scenario 1, is used to train the model.

#### Scenario 3

After the learning process in scenario 2, MEC spatiotemporal grid cells were inactivated to investigate how this affects the spatial firing of learnt hippocampal place cells after spatiotemporal input from MEC grid cells is lost. MEC grid cells are inactivated by setting the bottom 900 rows of **A** (the connection weights from MEC spatiotemporal grid cells to modelled hippocampal cells) to zero and then normalizing each column of **A** to have length 1. In this scenario, there is no learning.

The connection weight matrix **A** is initialized according to a uniform distribution between 0 and 1 and then normalized to have length 1 for each column. For both scenarios 1 and 2, a running trajectory of 3600 s is used to train the model and the connection weight matrix, **A**, is learnt during this training process. After learning, another running trajectory of 1200 s is used to recover the spatial receptive fields of these 100 modelled hippocampal cells for all three scenarios.

The parameters in Equations 4 and 6 that control the temporal properties of MEC grid cells are chosen as follows: *k_ϕ_* is chosen randomly from a uniform distribution between 0.8 and 1.2; *ϕ*_0_ and Δ*ϕ* are both chosen randomly from a uniform distribution between 300° and 340°; and *F* is 10 Hz.

For the parameters in the model dynamics and learning rule (Eqs. 7, 8), *τ* is 10 ms, *β* is 0.3, and the time step for simulating the modelled hippocampal cells by Equation 7 is taken to be 0.2 ms. Because the trajectory is updated after every 10 ms, there are 50 iterations of computing the response of modelled hippocampal cells using Equation 7. The connection weight matrix, **A**, is updated using Equation 8 after 50 iterations and the learning rate *η* is 0.01.

### Recovering the spatial receptive fields of modelled hippocampal cells

After training, another running trajectory of the virtual rat with a duration of 1200 s was used to recover the spatial receptive fields (rate maps) of modelled hippocampal cells. The 1m × 1m environment is discretized into a 40 × 40 lattice and the receptive field is recovered as the averaged response across all locations along the running trajectory of 1200 s.

#### Fitting the spatial receptive fields

In order to quantitatively characterize the spatial receptive fields of learnt hippocampal cells, the recovered receptive fields using the approach above is fitted by the following function:

(9) $${\hat{f}}_{s}(r)={\hat{\alpha }}_{c}e^{-\ln\;5\frac{||r-{\hat{r}}_{c}|{|}^{2}}{{\hat{R}}^{2}}},$$

where ${\hat{\alpha }}_{c}$ is the amplitude, ${\hat{r}}_{c}$ is the center and $\hat{R}$ determines the radius of the receptive field. The fitting routine is performed using the built-in MATLAB (version R2020a) function *lsqcurvefit*, and the fitting error is defined as the ratio between the summed square of the fitting residual and the summed square of the recovered receptive field.

#### Selecting place cells

The criteria for categorizing a modelled hippocampal cell as a place cell are: (1) the center, ${\hat{r}}_{c}$, is inside the spatial environment; (2) the radius, $\hat{R}$, is larger than 5 cm; and (3) the fitting error is smaller than 40%, where the fitting error is defined as the square of the ratio between the fitting residual and spatial field. The receptive field of a place cell is called the place field. These last two criteria are designed to preserve learnt hippocampal place cells that have a reasonable size of the place field and only one dominant place field. A modelled hippocampal cell that meets the criteria is referred to as “learnt hippocampal place cell.”

### Fitting the spatiotemporal response of learnt hippocampal place cells

The MEC spatiotemporal grid cells in the model have built-in spatial and temporal properties as described in Equations 2–4 and 6, so their response at a fixed position over 1 s displays the periodic pattern illustrated in Figure 1. However, modelled hippocampal cells have neither built-in spatial nor temporal properties. After learning, the response of modelled hippocampal cells is determined by the model dynamics (Eq. 7) where the connection weight matrix **A** is learnt during the virtual rat navigation. To investigate the temporal properties of learnt hippocampal place cells after learning, the response of learnt hippocampal place cells over 1 s at a given location is fitted to the following function:

(10) $$\hat{f}=\hat{\alpha }e^{{\hat{k}}_{\phi }\left(\cos(2\pi \hat{F}t-\hat{\phi })-1\right)},$$

where $\hat{f}$ denotes the fitted response, $\hat{\alpha }$ is the fitted amplitude that represents the spatial firing rate at this location, ${\hat{k}}_{\phi }$ is the estimated parameter that controls the level of dependence on phase precession, $\hat{F}$ is the estimated frequency of the response, and $\hat{\phi }$ is the estimated phase of the response. The fitting routine is performed using the built-in MATLAB (version R2020a) function *lsqcurvefit*. The fitting error is defined as the square of the ratio between the fitting residual and original response.

### Measuring the temporal properties of learnt hippocampal place cells

In order to quantitatively investigate the temporal properties of theta phase precession of learnt hippocampal place cells after learning, the following approach was used. First, for the learnt hippocampal place cells that meet the criteria of a place cell described earlier (see above, Selecting place cells), only cells whose entire place fields lie in the spatial environment are considered. Second, for each learnt hippocampal place cell, a virtual rat starts from the left side of the place field with an initial running rightwards direction and then a curved trajectory is generated according to Equation 1. Third, the response over 1 s at each position along the trajectory is generated by the model and then fitted using Equation 10. Finally, the entry phase, exit phase, and correlation coefficient between firing phases and normalized pdcd are obtained. For a learnt hippocampal place cell with theta phase precession, entry phase should be large and close to 360°, exit phase should be small and close to 0°, and the correlation coefficient between firing phases and normalized pdcd should be negative and close to −1.

### Code availability

The code of implementing the model and analyzing results was written in MATLAB (R2020a), and is available at https://github.com/yanbolian/Learning-Spatiotemporal-Properties-of-Hippocampal-Place-Cells.

## Results

### Scenario 1: the spatiotemporal properties of hippocampal place cells can be inherited from MEC grid cells via plasticity

**Figure 3.**
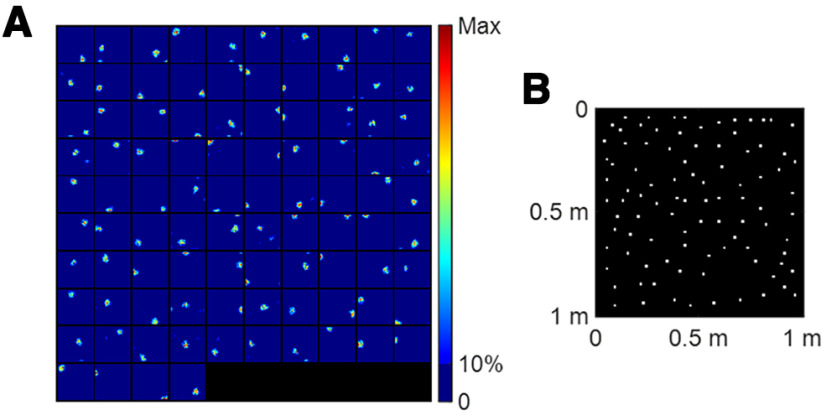
Scenario 1. Learnt hippocampal place cells display spatial properties. ***A***, Place fields of 94 (out of 100) learnt hippocampal place cells that satisfied the three criteria for a place cell. Each block represents the spatial receptive field (rate map) of a cell in a 1m × 1m environment. ***B***, Centers of these 94 learnt hippocampal place cells plotted together in the 1m × 1m environment. Values of parameters used in the simulation are given in Table 1.

The results presented here are those from scenario 1, namely, the simulation in which only MEC spatiotemporal grid cells provide input for the modelled hippocampal cells. The MEC spatiotemporal grid cell to hippocampal cell connectivity was learnt using non-negative sparse coding over 3600 s of virtual rat navigation over the 1m × 1m environment, as described in Materials and Methods, Training. Through this learning, the learnt hippocampal place cells learn to pool different MEC spatiotemporal grid cells in such a way that they become selective to specific locations in the spatial environment. The receptive fields of the 94 out of 100 modelled hippocampal cells that meet the criteria for place cells described in Materials and Methods, Selecting place cells, are plotted in Figure 3*A*. These learnt hippocampal place cells have a dominant firing location and their firing locations differ from each other. The centers of each of these 94 place fields are displayed in Figure 3*B*, which are estimated using Equation 9. This figure shows that the population of 94 learnt hippocampal place cells tile the entire spatial environment in a fairly uniform fashion. Compared with our previous work (Lian and Burkitt, 2021, see their Fig. 3), the tiling of hippocampal place cells here is less uniform because of the lack of uniformity in the input spatial locations: the spatial locations in Lian and Burkitt (2021) are randomly sampled from a uniform distribution while the spatial locations in this paper are generated by a rat running trajectory described in Equation 1.

**Figure 4.**
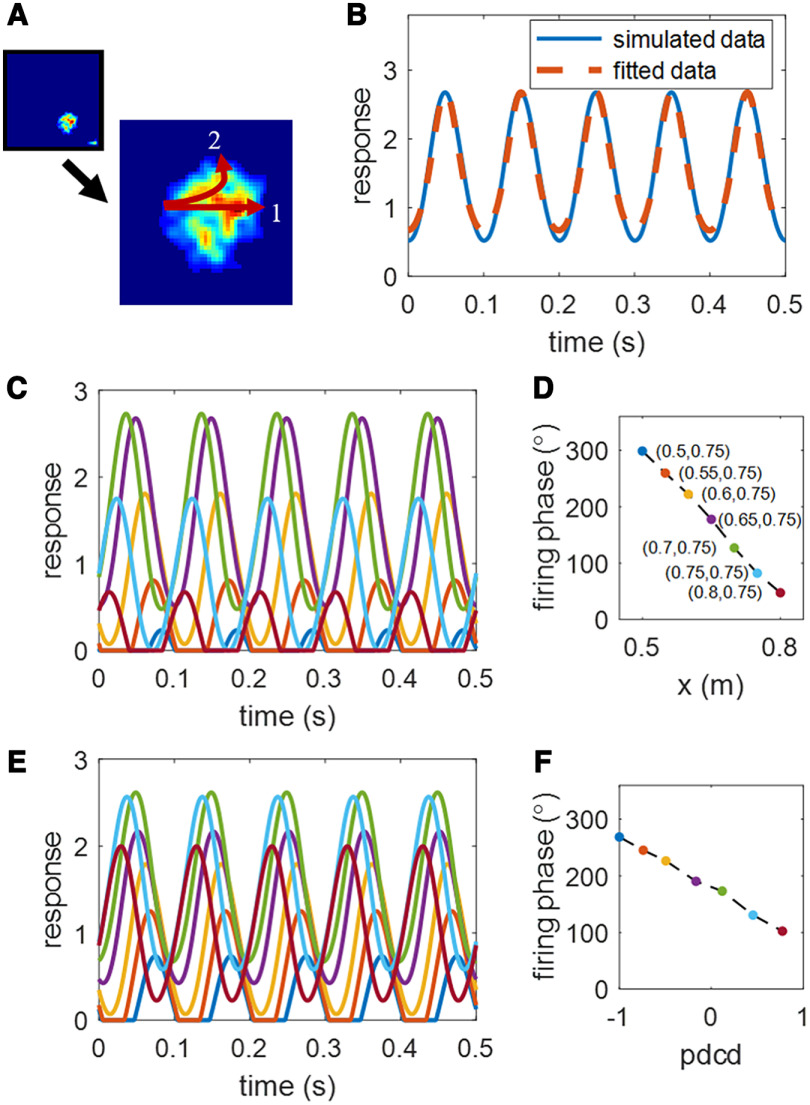
Scenario 1. One example of learnt hippocampal place cell’s temporal response properties. ***A***, Rate map of an example of learnt place cells whose center is at (0.65, 0.75). ***B***, The response (solid line) over 0.5 s at the center of the place field and the fitted response (dashed line) by Equation 10. ***C***, The curves in different colors represent responses of this learnt hippocampal place cell over 0.5 s at different locations (coordinates found in plot ***D*** with the same color coding) along the straight trajectory 1 in plot ***A***. ***D***, Estimated firing phase, $\hat{\phi }$, versus position. Only *x* varies because the virtual rat moves from left to right. ***E***, The curves in different colors represent responses of this learnt hippocampal place cell over 0.5 s at different locations (normalized pdcd found in plot ***F*** with the same color coding) along the curved trajectory 2 in plot ***A***. ***F***, Estimated firing phase, $\hat{\phi }$, versus normalized pdcd. Values of parameters used in the simulation can be found in Table 1.

Furthermore, these learnt hippocampal place cells also display the temporal property of theta phase precession. This is illustrated in the response of a learnt hippocampal place cell over 0.5 s shown in Figure 4.

Figure 4*B* shows the response of one learnt hippocampal place cell over 0.5 s at position (0.65, 0.75), which is the center of the place field shown in Figure 4*A*. The solid line in Figure 4*B* represents the response of this example place cell over 0.5 s and displays a periodic pattern. After fitting the response of the learnt place cell to the spatiotemporal function described by Equation 10, the fitted response is plotted in the dashed line. The response of this learnt place cell is well fitted by Equation 10 and the fitting error is small (0.35%). The fitted parameters are $\hat{\alpha }=2.68,\;{\hat{k}}_{\phi }=0.69,\;\hat{F}=10.00$ Hz and $\hat{\phi }=177.77^{{}^{\circ}}$.

The response of the same example place cells over 0.5 s as the animals runs from left to right of the place field (through the field center) along the straight trajectory 1 in Figure 4*A* is shown in Figure 4*C*. As the virtual rat is at position (0.5, 0.75), the amplitude of the response curve is below 1 and the firing phase (indicated by the peak location) is close to the end of each cycle (i.e., late phase). As the virtual rat continues running to the right, the amplitude of the response curve increases and then decreases, while the firing phase continues to shift forward to the beginning of each cycle. After fitting the response curve by Equation 10, the relationship between firing phase and position is shown in Figure 4*D*, which shows a clear reverse correlation, namely, that the firing phase moves from late phase to early phase as the virtual rat crosses the place field. The entry phase and exit phase are 303.9° and 28.7°, respectively. The correlation coefficient between firing phases and positions is –0.998. Therefore, as the virtual rat runs from the left to the right of the place field, this place cell response displays theta phase precession. In other words, after learning, the resulting hippocampal place cells not only display spatial place fields in their responses but also display phase precession.

The learnt place cell also displays theta phase precession when the virtual rat crosses the place field along the curved trajectory 2 in Figure 4*A*, as illustrated in Figure 4*E*,*F*. Since in this case both *x* and *y* values of the position change, we plot the firing phase relative to the normalized pdcd (similar to Jeewajee et al., 2014) instead of positions. The amplitude of the response curve is small when the virtual rat enters the place field, increases to the peak, and then decreases as the virtual rat leaves the place field. The firing phase is observed to shift from late phase to early phase, i.e., displaying theta phase precession, similar to the straight trajectory but with a somewhat narrow range of phases. The entry phase is 268.8°, exit phase is 102.3°, and the correlation coefficient between firing phases and normalized pdcd is –0.998.

Consequently, the example place cell in Figure 4 learns both spatial and temporal properties after training. These spatiotemporal properties for the learnt hippocampal place cells are not inherent properties of the place cells, but arise entirely because of the way in which the model learns a place-specific tuning and theta phase precession from the spatiotemporal information provided by MEC grid cells.

**Figure 5.**
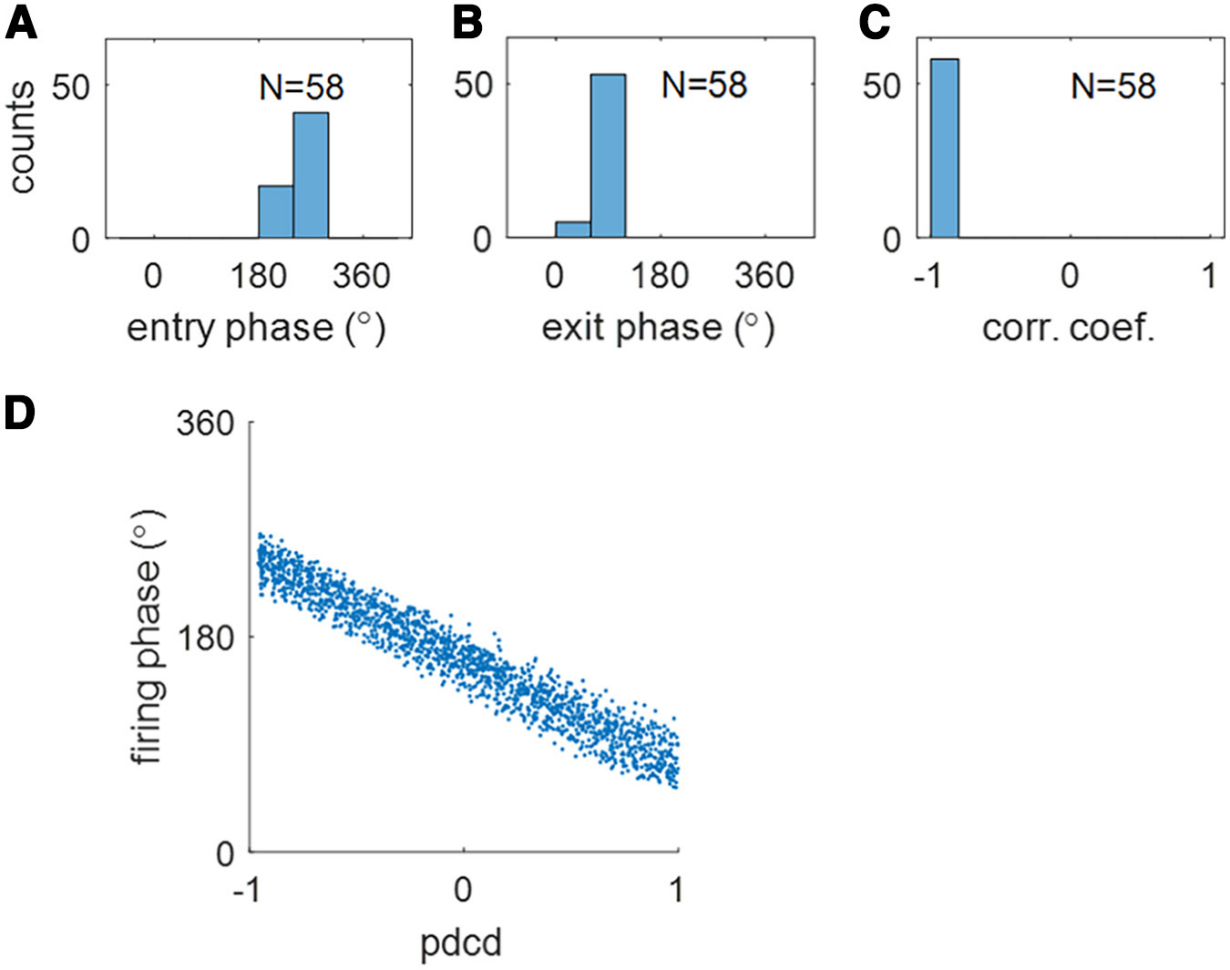
Scenario 1. The population of learnt hippocampal place cells displays temporal properties. Histograms of (***A***) the entry phases, (***B***) the exit phase, and (***C***) the correlation coefficient show that learnt hippocampal place cells have strong theta phase precession. ***D***, Scatter plot of firing phase, $\hat{\phi }$, versus pdcd, clearly showing theta phase precession of learnt hippocampal population of cells. Values of parameters used in the simulation are given in Table 1.

Among the 94 (out of 100) learnt hippocampal place cells, 58 place cells have their entire place fields inside the spatial environment. For each place cell, a random curved trajectory is generated that crosses the place field and the neuronal response is computed at different locations along the trajectory (see Materials and Methods, Measuring the temporal properties of learnt hippocampal place cells). The population statistics of the temporal properties are shown in Figure 5. Figure 5*A*,*B* show that the firing phase of these cells is at a late phase when the virtual rat enters the place field and at an early phase when the virtual rat leaves the place field. The histogram of correlation coefficients between firing phase and normalized pdcd (Fig. 4*C*) and scatter plot of firing phase and normalized pdcd (Fig. 4*D*) demonstrate strong theta phase precession. Recall that MEC grid cells are constructed here to have this built-in phase precession property (characterized by Eqs. 4, 6). For each grid field of MEC grid cell, the firing phase and normalized pdcd along any trajectory are constructed to have a correlation coefficient of −1 because of the linear relationship between the firing phase and pdcd (Eq. 6). Both the spatial and temporal response properties of hippocampal place cells are entirely learnt from pooling the input they receive from MEC grid cells. Furthermore, the fact that the correlation coefficients in Figure 5 are very close to −1 indicates that theta phase precession is well preserved in the learnt hippocampal place cells.

### Scenario 2: the spatiotemporal properties of hippocampal place cells can be learnt when both MEC grid cells and EC weakly spatial cells serve as the EC input to the hippocampus

In this section, we show the results of training with scenario 2, namely, where both MEC spatiotemporal grid cells and EC weakly spatial cells provide input to the modelled hippocampal cells. The results show that both MEC grid cells and EC weakly spatial cells provide spatial information for the hippocampus, so that a spatial hippocampal map can be retrieved from upstream input, while MEC grid cells provide temporal information that results in theta phase precession also being inherited from the upstream neural population.

**Figure 6.**
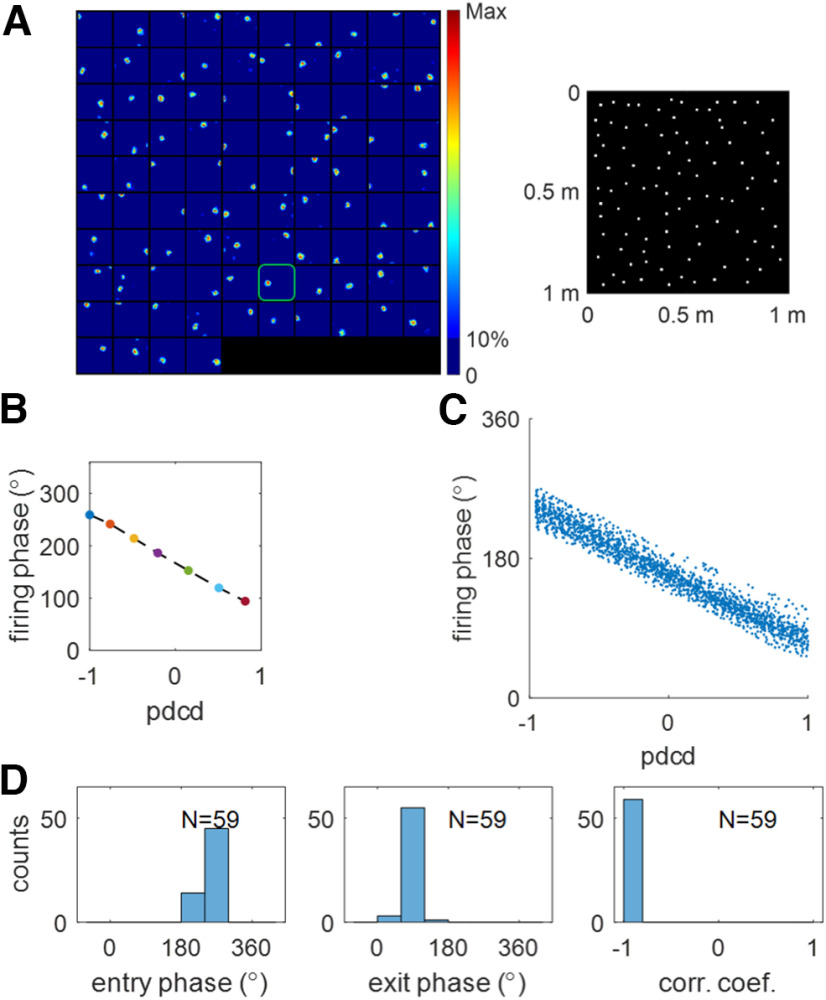
Scenario 2. Learnt hippocampal place cells display spatiotemporal properties. ***A***, Spatial properties of learnt hippocampal place cells. Left, The place fields of learnt hippocampal place cells. Right, Place field centers plotted in the same environment. ***B***, Firing phase, $\hat{\phi }$, versus normalized pdcd along a curved trajectory that crosses the place field, which illustrates theta phase precession for one learnt hippocampal place cell highlighted in plot ***A***. ***C***, The scatter plot of firing phase, $\hat{\phi }$, versus pdcd, clearly showing theta phase precession of learnt hippocampal population. ***D***, The population of learnt hippocampal place cells displays temporal properties. Histograms of entry phase (left), exit phase (middle), and correlation coefficient (right) indicate a strong phase precession for the population of learnt hippocampal place cells. Values of parameters used in the simulation are given in Table 1.

A demonstration of how EC weakly spatial cells and MEC grid cells contribute to the spatial and temporal properties of hippocampal place cells is given in Figure 6. After learning, the recovered rate maps show place-field like receptive fields. As seen from Figure 6*A*, different hippocampal place cells learn different place fields and the hippocampal population tiles the entire environment, similar to scenario 1 in which only MEC grid cells provide input to the hippocampus (Fig. 3). An example of the temporal response properties of a place cell can be seen from the relationship between firing phase and normalized pdcd within the place field in Figure 6*B*, which displays characteristic theta phase precession: as the virtual rat moves along a random generated curved trajectory (see Materials and Methods, Measuring the temporal properties of learnt hippocampal place cells), the firing phase shifts smoothly from a late phase to an early phase. Figure 6*C*,*D* displays the population statistics for this phase precession, similar to Figure 5, indicating that learnt hippocampal place cells display strong phase precession.

### Scenario 3: spatial properties of hippocampal place cells are maintained after the inactivation of MEC grid cells

After the learning process in scenario 2, MEC spatiotemporal grid cells in the model were inactivated, i.e., only the remaining EC weakly spatial cells provide spatial information for hippocampal place cells and there is no input (temporal or spatiotemporal) from MEC grid cells. Recall that there is no learning in scenario 3, so the spatial tuning of hippocampal place cells depends on the connectivity between EC weakly spatial cells and hippocampal place cells that was learnt in scenario 2.

**Figure 7.**
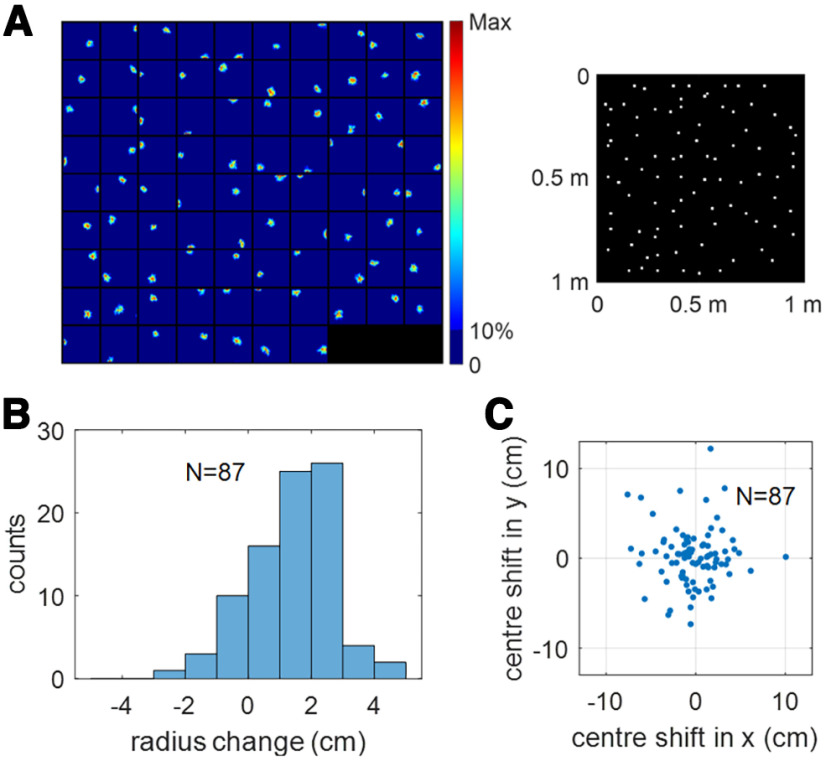
Scenario 3. Learnt hippocampal place cells maintain spatial firing after MEC inactivation. ***A***, The place fields of 87 hippocampal place cells (left) and their centers plotted in the same environment (right). ***B***, Histogram of radius change of place fields after MEC inactivation. The radius of most place fields increases after MEC inactivation. The mean radii before and after MEC inactivation are 8.94 and 10.34 cm, respectively. ***C***, Scatter plot of the place field center shift after MEC inactivation.

However, the learnt connection between EC weakly spatial cells and place cells is sufficient to maintain the place field of hippocampal place cells after MEC inactivation. After recovering the receptive fields of modelled hippocampal cells (see Materials and Methods, Recovering the spatial receptive fields of modelled hippocampal cells), 87 out of 100 modelled hippocampal cells were found to meet the criteria of a place cell. The place fields, together with their field centers, are plotted in Figure 7*A*. Comparison with Figure 6*A* shows that the place fields still maintain their firing locations and the population still tiles the entire spatial environment. This indicates that the learnt connection from EC weakly spatial cells to place cells during free running provides sufficient spatial information, such that stable place fields will not be lost although MEC grid cells are inactivated in this scenario.

There are 94 place cells before inactivating MEC grid cells (Fig. 6*A*, scenario 2) and 87 place cells after the inactivation (Fig. 7*A*, scenario 3). We find that all these 87 place cells that pass the criteria for place cells after MEC inactivation (scenario 3) come from the population of 94 place cells before MEC inactivation (scenario 2). After inactivating MEC grid cells, Figure 7*B* shows that, in general, the size of the place fields increases. The mean radii before and after MEC inactivation are 8.94 cm and 10.34 cm, respectively. The increase of place field size after MEC activation is consistent with experimental results (Schlesiger et al., 2015). Furthermore, although most place cells maintain stable place fields after the inactivation, their field centers shift randomly within a small range, as seen in Figure 7*C*. These results also suggest that the place coding of the hippocampus becomes somewhat less accurate after MEC inactivation.

This result is consistent with the experimental study by Schlesiger et al. (2015) who found that after MEC lesions in rats, theta phase precession of hippocampal CA1 cells was significantly disrupted, although stable spatial firing was maintained. Although the loss of excitatory drive from MEC grid cells has reduced the spatial accuracy of the place cells, spatial properties can nevertheless be somewhat maintained by the excitatory drive from other cells such as weakly spatial cells in the MEC. This reduced spatial accuracy because of the loss of the MEC excitatory drive has been interpreted by some authors as an impaired information flow to the hippocampus, because of the role of neuronal oscillations in the coding of spatial information (Brandon et al., 2014). Therefore, during the navigation of the virtual rat in scenario 2, the plasticity of the model allows hippocampal place cells to pool the input from upstream neurons, namely, the EC weakly spatial cells or MEC grid cells, enabling both spatial and temporal properties to be learnt.

Combining results in this paper and our previous work (Lian and Burkitt, 2021), the contribution of the EC to the firing of hippocampal place cells can be described as follows: both EC weakly spatial cells and cells with a clear structure (such as MEC grid cells) provide spatial information for the hippocampus to learn an efficient hippocampal place map, while the temporal response properties involving theta phase precession of hippocampal place cells are inherited from MEC grid cells via learning during navigation.

## Discussion

### Summary

In this study, a model based on sparse coding is built that demonstrates that the spatial and temporal properties of hippocampal place cells can be learnt simultaneously via plasticity as the virtual rat freely explores an open environment. In the training phase, a virtual rat runs for 3600 s and the connectivity weight matrix, **A**, is learnt during this exploration period. After learning, **A** is kept fixed and another running trajectory of 1200 s is used to recover the place fields of learnt place cells. In addition, responses over 1 s at different positions across the place field are used to investigate the firing phases when the virtual rat traverses the place field. Similar to the study of Jeewajee et al. (2014), pdcd is used as the measurement of position when the rat is running along a curved trajectory. Our results show that the learnt hippocampal place cells are selective to a single firing location and display theta phase precession, although the learnt hippocampal place cells have no prebuilt spatial and temporal properties. Furthermore, the model shows that the loss of MEC grid cells causes the loss of the temporal response properties of hippocampal place cells, but the spatial properties of hippocampal place cells are maintained by other upstream cells, such as EC weakly spatial cells, that provide spatial information for the hippocampus. This model demonstrates how the spatiotemporal properties of hippocampal place cells can be learnt via synaptic plasticity.

### Comparison with other models of temporal properties of place cells

Our model demonstrates that the temporal properties of hippocampal place cells can be inherited from MEC grid cells via synaptic learning. However, there are also other models that describe the temporal properties of place cells from other perspectives.

O’Keefe and Recce (1993) proposed that the temporal properties of place cell can be modelled by single neuron properties and may originate from the interference between two intrinsic oscillators of slightly different frequencies: a slower baseline oscillation that generates the local field potential of hippocampal theta rhythm and a relatively faster oscillation of place cells when the animal moves in the place field. Supposing that the slower oscillation and faster oscillation are synchronized at the beginning, then over time the peak of the faster oscillation will shift forward compared with the baseline oscillation. When the place cell fires at the peak of the faster oscillation, then the firing phase relative to the slower baseline oscillation will shift to an earlier phase, which is the well-known theta phase precession. Additionally, the difference between the frequencies of the slower and faster oscillations is proportional to running speed, so the firing phase will reflect the distance traveled through the place field. This model has led to numerous related models with similar dynamics (Kamondi et al., 1998; Bose et al., 2000; Harris et al., 2002; Mehta et al., 2002; Lengyel et al., 2003).

The intrinsic oscillator mechanism explains how phase precession operates on the basis of single neurons, but hippocampal phase precession can also be explained by a network mechanism in which the interaction between neurons in the network plays a crucial role. Tsodyks et al. (1996) built a spiking network of hippocampal place cells that receives intrinsic input from neurons in the network and an extrinsic theta rhythm from the medial septum. The synaptic interaction between neurons in this model network is asymmetric, namely, the synaptic strengths aligned with the motion direction are stronger than those aligned in the direction opposite to the motion. This asymmetric connectivity brings some degree of direction tuning of the model place cells in the network, so the firing of place cells becomes successively earlier relative to the theta rhythm, thereby causing theta phase precession. Although the asymmetric connectivity in the study by Tsodyks et al. (1996) was manually set, this model is supported by experimental evidence that shows that hippocampal place fields are experience-dependent and can become asymmetric during route following (Mehta et al., 1997). In addition, this asymmetry can be generated by Hebbian plasticity, as supported by modeling studies that demonstrate how such asymmetric connectivity can be learnt via long-term potentiation/long-term depression (Mehta et al., 2000) or spike-timing-dependent plasticity (Burkitt and Hogendoorn, 2021) in an environment where the motion in the same direction is repeated many times. Apart from Hebbian plasticity, the asymmetric connectivity of the network may also arise from other sources: (1) it may arise during the developmental process, as supported by experimental studies which demonstrate that hippocampal place cells fire in sequences even before the exploration of a novel environment (Dragoi and Tonegawa, 2011); (2) it may be preconfigured as suggested by Mizuseki and Buzsáki (2013).

Both the intrinsic oscillator mechanism and network mechanism require a baseline theta rhythm as the input, but the two mechanisms differ in how the firing phase shifts relative to the baseline oscillation: one is caused by the faster oscillation of single place cell and the other is caused by the interaction between place cells in the network. However, this does not imply that these two mechanisms are completely contradictory. Instead, they can be unified within a perspective that hippocampal place cells require both external input (extrinsic) and local interaction (intrinsic) to achieve spatial-temporal properties, as supported by a recent experimental study (Zutshi et al., 2022).

Experimental studies indicate that phase precession may not originate in the hippocampus, but rather it is inherited from the upstream processing (Burgess et al., 1994; Skaggs et al., 1996; Zugaro et al., 2005; Schlesiger et al., 2015). In addition, Zutshi et al. (2022) demonstrate that the MEC is the main current generator of hippocampal theta oscillations.

The work presented in this paper models temporal properties of hippocampal place cells as being inherited from upstream processing, as proposed by earlier studies (Skaggs et al., 1996; Hafting et al., 2008; Bush et al., 2014; Schlesiger et al., 2015). Although there are other models of hippocampal phase precession based on the inheritance of upstream (Yamaguchi et al., 2007; Jaramillo et al., 2014), our modeling work show that this inheritance of temporal properties comes together with the observed spatial properties via a form of synaptic plasticity.

Our model is also related to the intrinsic oscillator mechanism and the extrinsic oscillator mechanism because it requires oscillatory input and local interactions between cells in the network: the oscillatory input comes from the MEC grid cells that have temporal response properties and the local interactions between cells introduce competition (sparsity) into the network such that cells learn different representations. Experimental studies suggest that the projections from medial septal areas also control hippocampal oscillations (Bender et al., 2015; Dannenberg et al., 2015; Zutshi et al., 2018; Quirk et al., 2021), but our current model does not take direct oscillatory input from medial septum. In addition, although our model utilized local computation within the network, it cannot account for the self-organized dynamics of hippocampal networks in its current form.

Essentially, our model provides a learning framework in which spatial-temporal properties of hippocampal place cells can be inherited from MEC via synaptic learning. This framework may interact with other mechanisms to help us better understand the hippocampus.

### The entorhinal-hippocampal loop

Early computational models of place cells were mostly based on a feedforward structure, where cells in the EC provide spatial input to the hippocampus (Solstad et al., 2006; Franzius et al., 2007a,b; de Almeida et al., 2009). Some more recent studies have adopted a loop network structure in which cells in the EC project to the hippocampus and also receive feedback from it (Rennó-Costa and Tort, 2017; Agmon and Burak, 2020; Li et al., 2020). Models incorporating this loop network structure can explain additional experiment observations, especially concerning how hippocampal place cells affect the firing of MEC grid cells. Since the hippocampus receives information from different brain areas via the EC, place cells in the hippocampus can still maintain some properties even when MEC grid cells are inactivated. On the other hand, hippocampal place cells also affect the grid pattern of MEC grid cells (Bonnevie et al., 2013; Almog et al., 2019). Therefore, the feedback from the hippocampus to the EC can modify some properties of EC cells, which can only be investigated using the loop network structure. However, the results presented here do not rule out the possibility of implementing the model in a loop network structure because sparse coding can be implemented in a feedforward-feedback loop (Lian et al., 2019).

### Evidence against feedforward grid-to-place models

Recent research has provided experimental evidence that place fields emerge earlier in development than MEC grid cells (Langston et al., 2010; Wills et al., 2010). There is also experimental evidence for the maintenance of stable place fields after the inactivation of MEC grid cells (Brandon et al., 2014; Hales et al., 2014; Schlesiger et al., 2015). These results put into question the feedforward nature of grid-to-place cell models. However, unlike typical grid-to-place cell models, our model takes input from different types of cells in the EC. The results presented in this paper, together with our previous work (Lian and Burkitt, 2021), provides a unifying framework that is able to explain this experimental evidence while supporting a hierarchical structure of connectivity from the EC to the hippocampus in which theta phase precession of hippocampal place cells is essentially inherited from MEC gird cells via learning.

Bonnevie et al. (2013) showed that the inactivation of place cells will cause the loss of MEC grid cells. However, Almog et al. (2019) re-analyzed the same data and found that grid cells maintain synchrony although grid tuning is lost. In addition, a large number of grid cells actually maintain spatial tuning after the reactivation of place cells (Almog et al., 2019, see their Fig. 1D). Therefore, although hippocampal place cells affect the spatial firing of some MEC grid cells, this does negate the importance of the feedforward connection from the EC to the hippocampus. A future model that can capture the contribution of the EC to the hippocampus as well as the effect that the hippocampus has on the EC is needed.

### Future work

While this study offers a picture of how the EC contributes to the spatial and temporal properties of hippocampal place cell, there remain a number of interesting outstanding questions to be addressed. First, the question of how the feedback from the hippocampus to the EC affects properties of EC cells remains unclear. Second, the model proposed here demonstrates how sparse coding can learn spatiotemporal properties of hippocampal place cells, but whether the principle of sparse coding can be used to explain other aspects of hippocampal function, such as those involving memory consolidation, remains unclear. In addition, the spatiotemporal model of MEC grid cells is assumed here, and the question of how this MEC spatiotemporal grid cell structure originates is an active topic of ongoing research. Moreover, the model presented here uses rate-based neurons, and a spiking implementation together with spike-timing dependent plasticity of this model is left for future research.

## Synthesis

Reviewing Editor: Michaël Zugaro, CNRS, Collège de France, Inserm Decisions are customarily a result of the Reviewing Editor and the peer reviewers coming together and discussing their recommendations until a consensus is reached. When revisions are invited, a fact-based synthesis statement explaining their decision and outlining what is needed to prepare a revision will be listed below. The following reviewer(s) agreed to reveal their identity: NONE.

The manuscript presents a careful and well planned theoretical investigation of how hippocampal theta phase precession could be learned from afferent regions of the temporal lobe. CA1 pyramidal neurons would acquire spatial and temporal properties via synaptic plasticity. It considers a detailed model of grid cell activity based on the separation between its rate coding and phase coding components, a somewhat less detailed model of the activity of other entorhinal cells (“weakly spatial”) that are assumed to convey additional inputs to the place cells, and a model of synaptic plasticity, based on the notion of non-negative sparse coding, elegantly formulated in matrix form. It confirms that the hypothesized mechanism can work (whether weakly spatial cells are included or not, the latter not contributing to phase precession). Phase precession in place cells then survives if grid cells are inactivated.

The main caveat is that the model is a purely descriptive model that tries to capture in simplified mathematical form a natural phenomenon, rather than to explain it. Perhaps not so surprisingly, the model manages to confirm that phase precession could arise in place cells, given that it occurs in grid cells. Still, this had not been directly shown yet, and the manuscript appears suitable for publication in eNeuro, provided the findings are presented in a broader context.

Indeed, several models of phase precession have been proposed over the years, in particular from the McNaughton lab (Skaggs, Wilson, Mehta), even before grid cells were discovered. The phenomenon may be so easy to produce as to be present, in disguise, even in the absence of genuine theta oscillations (e.g. in bats). This and other pertinent research should be discussed. In particular, an alternative framework is based on the observation that network asymmetries will favor propagation of activity, as described by Tsodyks et al. (1996; PMID: 8841826). Such asymmetries could arise during development: this is supported by the finding that neurons already fire in sequences before the exploration of a novel environment (Dragoi and Tonegawa, 2011; PMID: 21179088), as well as by the log-normal distribution of activity within the hippocampus, which is consistent with the idea that networks could be at least partially pre-configured (Mizuseki & Buzsaki, 2013; PMID: 23994479). From this perspective, spatial and temporal properties may not result from Hebbian plasticity, but from network pre-configuration (synaptic strength determined throughout development and via learning). This alternative framework should be discussed.

As a side note, previous studies investigating the properties of mEC or hippocampal neurons following their respective inactivation have interpreted the respective effects in terms of impaired ‘information ’ flow. Yet, this could be more appropriately described as a loss in excitatory drive (while both CA1 place cells and grid cells are impaired following septal inactivation, the septum is rarely if ever described as an information conduit). This caveat should be mentioned and discussed.

## Author Response

Dear eNeuro Editor,

Please find attached the response to the reviews of our manuscript, “Learning spatio-temporal properties of hippocampal place cells” that was submitted to eNeuro (eN-TNWR-0519-21). The reviews, which are very constructive, have helped to improve the paper and provide greater impact of our work to the community.

In response to the reviewer comments, we have revised the manuscript to improve the readability of the paper, connect our results more closely with the existing literature and highlight the novel aspects of the work. We have also given more emphasis to the results on obtaining an efficient hippocampal place map using weakly spatial cells, which provides another possibility for the formation of place cells.

In response to the comments of Reviewers:

1. The authors provide a link to the code.

• Response: We added a section ‘Code availability’ to the methods section: “The code of implementing the model and analyzing results was written in MATLAB (R2020a, USA), and is available at https://github.com/yanbolian/Learning-Spatiotemporal-Properties-of-Hippocampal-Place-Cells.”

2. Indeed, several models of phase precession have been proposed over the years, in particular from the McNaughton lab (Skaggs, Wilson, Mehta), even before grid cells were discovered. The phenomenon may be so easy to produce as to be present, in disguise, even in the absence of genuine theta oscillations (e.g. in bats). This and other pertinent research should be discussed. In particular, an alternative framework is based on the observation that network asymmetries will favor propagation of activity, as described by Tsodyks et al. (1996; PMID: 8841826). Such asymmetries could arise during development: this is supported by the finding that neurons already fire in sequences before the exploration of a novel environment (Dragoi and Tonegawa, 2011; PMID: 21179088), as well as by the log-normal distribution of activity within the hippocampus, which is consistent with the idea that networks could be at least partially pre-configured (Mizuseki & Buzsaki, 2013; PMID: 23994479). From this perspective, spatial and temporal properties may not result from Hebbian plasticity, but from network pre-configuration (synaptic strength determined throughout development and via learning). This alternative framework should be discussed.

• Response: We add a section ‘Comparison with other models of temporal properties of place cells’ to the discussion section: “Our model demonstrates how the temporal properties of hippocampal place cells can be inherited from grid cells via synaptic learning, but there are also other models of temporal properties of place cells from other perspectives.

O’Keefe and Recce (1993) proposed that the temporal properties of place cell may originates from the interference between two oscillators of slightly different frequencies, and this has led to numerous related models with similar dynamics (Kamondi et al., 1998; Bose et al., 2000; Mehta et al., 2002; Harris et al., 2002; Lengyel et al., 2003). Tsodyks et al. (1996) built a spiking network of hippocampal place cells that receives intrinsic input from neurons in the network and an extrinsic theta rhythm from the medial septum. The synaptic interaction between neurons in this model network is asymmetric, which brings some degree of direction tuning of the model place cells in the network, so it causes theta phase precession. This model is supported by experimental evidence that hippocampal place fields are experience-dependent and can become asymmetric during route following (Mehta et al., 1997), and that this asymmetry may also come from the pre-configured hippocampal network (Mizuseki and Buzs aki, 2013) or from the developmental process (Dragoi and Tonegawa, 2011).

The work presented in this paper models temporal properties of hippocampal place cells as being inherited from upstream, as proposed by other studies (Skaggs et al., 1996; Hafting et al., 2008; Bush et al., 2014; Schlesiger et al., 2015), and that this inheritance of temporal properties comes together with the observed spatial properties via learning.”

3. As a side note, previous studies investigating the properties of mEC or hippocampal neurons following their respective inactivation have interpreted the respective effects in terms of impaired ‘information’ flow. Yet, this could be more appropriately described as a loss in excitatory drive (while both CA1 place cells and grid cells are impaired following septal inactivation, the septum is rarely if ever described as an information conduit). This caveat should be mentioned and discussed.

• Response: We did the following to incorporate this comment.

o In Scenario 3 of Section 2.5, we added “Schlesiger et al. (2015) found that theta phase precession of hippocampal place cells was greatly disrupted by MEC lesions but hippocampal spatial firing still remained. This scenario is also similar to another experimental study that showed the retention of hippocampal place fields during medial septum inactivation (Brandon et al., 2014), which is presumed to shut off grid cell input due to the loss of excitatory drive (Koenig et al., 2011; Brandon et al., 2011).”

o In Section 3.3, we added “Though the loss of excitatory drive from MEC grid cells has reduced the spatial accuracy of the place cells, spatial properties can nevertheless be somewhat maintained by the excitatory drive from other cells such as weakly spatial cells in the MEC. This reduced spatial accuracy due to the loss of the MEC excitatory drive has been interpreted by some authors as an impaired information flow to the hippocampus, due to the role of neuronal oscillations in the coding of spatial information (Brandon et al., 2014).”

To incorporate the changes suggested by the reviewers, the manuscript has been significantly modified. The following list details the changes made in the manuscript:

Material and Methods

Add a section ‘Code availability’

Add a sentence in 2.5.

Results

Add some sentences to 3.3

Discussion

Add a section ‘Comparison with other models of temporal properties of place cells’

Thank you for your consideration and we look forward to hearing from you.

Yours sincerely,

Yanbo Lian

On behalf of the authors: Yanbo Lian, Anthony N. Burkitt
